# Antagonistic TGR5 and FXR Signaling Shapes STAT1-Dependent Interferon Responses in Intestinal Epithelial Cells

**DOI:** 10.3390/cells15201903

**Published:** 2026-10-20

**Authors:** Huilin Jia, Nannan Wu, Yufeng Yan, Fanzhi Kong

**Affiliations:** College of Animal Science and Veterinary Medicine, Heilongjiang Bayi Agricultural University, No. 5 Xinfeng Road, Sartu District, Daqing 163319, China; huilinjia@byau.edu.cn (H.J.); wunannan0420@byau.edu.cn (N.W.); yanyufeng030314@163.com (Y.Y.)

**Keywords:** bile acids, TGR5, FXR, STAT1, IFNλ3, ISG15, intestinal epithelial cells

## Abstract

Bile acids function not only as digestive metabolites but also as signaling molecules that influence mucosal immunity. However, how the bile acid receptors Takeda G-protein-coupled receptor 5 (TGR5; also known as GPBAR1) and farnesoid X receptor (FXR) coordinate intestinal epithelial interferon (IFN) responses remains incompletely understood. Here, we investigated the impact of TGR5 and FXR on IFN signaling in porcine IPEC-J2 and murine MODE-K intestinal epithelial cells. Pharmacological activation of TGR5 with INT-777 induced type I and type III IFNs together with multiple interferon-stimulated genes (ISGs), among which IFNλ3 and ISG15 showed the most prominent responses. Mechanistically, TGR5 enhanced STAT1 phosphorylation and nuclear accumulation, and both STAT1 silencing and pharmacological blockade of STAT1 phosphorylation attenuated TGR5-driven IFNλ3 and ISG15 expression. Inhibition of adenylate cyclase or protein kinase A further placed a cAMP-PKA-STAT1 axis downstream of TGR5. By contrast, activation of FXR with GW4064 suppressed poly(I:C)-induced IFNλ3 and ISG15 transcription and antagonized TGR5-mediated STAT1 activation and downstream gene expression. Confocal imaging further suggested that FXR restrains STAT1 nuclear trafficking through cytoplasmic colocalization with STAT1. Collectively, our findings identify a bile acid receptor rheostat in which TGR5 amplifies, whereas FXR dampens, epithelial IFN signaling through STAT1. This work provides a mechanistic framework for understanding how bile acid sensing may tune antiviral readiness at the intestinal mucosa.

## 1. Introduction

Interferons (IFNs) are central effectors of innate immunity and are rapidly induced when host cells sense pathogen-associated molecular patterns through pattern-recognition receptors [1,2]. Activation of these pathways drives the expression of type I, type II, and type III IFNs, which subsequently engage cognate receptors and trigger Janus kinase-signal transducer and activator of transcription (JAK-STAT) signaling to establish antiviral and immunoregulatory programs [3,4]. Among these pathways, signal transducer and activator of transcription 1 (STAT1) is a key transcriptional hub because its phosphorylation and nuclear translocation are required for the induction of numerous IFN-responsive genes [4,5]. Within the intestinal epithelium, type I and, especially, type III IFNs are critical for frontline antiviral defense [6,7]. Type III IFNs act in a more tissue-restricted manner than type I IFNs and are particularly important at epithelial barriers, where they limit viral replication while helping preserve barrier integrity [8,9,10,11]. IFN-driven induction of interferon-stimulated genes (ISGs), including ISG15, oligoadenylate synthase (OAS) family members, interferon-induced transmembrane (IFITM) proteins, and MX proteins, is a core component of this defense system [12,13]. Among these ISGs, ISG15 has emerged as a prominent epithelial antiviral effector and immunomodulatory molecule [14,15].

Bile acids are increasingly recognized as immunoregulatory metabolites in addition to their canonical roles in lipid digestion and absorption [16]. Accumulating evidence indicates that bile acids can influence antiviral immunity directly or indirectly through receptor-mediated signaling [17]. Two major bile acid receptors are the membrane G protein-coupled receptor TGR5 (also termed GPBAR1) and the nuclear receptor farnesoid X receptor (FXR) [17,18]. Both receptors are expressed in intestinal tissues and are activated by bile acid species with distinct ligand preferences and downstream signaling outputs [17]. Prior studies have shown that bile acids such as chenodeoxycholic acid, deoxycholic acid, lithocholic acid, and hyodeoxycholic acid can modulate epithelial antiviral responses, and that TGR5 or FXR may contribute to these effects [19,20,21].

Our previous work showed that bile acids increase IFN-β, IFNλ3, and ISG15 expression in porcine intestinal epithelial cells and suppress porcine deltacoronavirus replication, whereas other studies have further implicated TGR5 in bile acid-driven epithelial antiviral signaling [22]. Conversely, recent evidence suggests that FXR can dampen interferon signaling and thereby weaken the antiviral efficacy of bile acids [20]. Despite these observations, the molecular basis by which TGR5 and FXR exert opposing effects on epithelial IFN responses remains insufficiently defined. In the present study, we used porcine and murine intestinal epithelial cell models to dissect how TGR5 and FXR regulate IFN signaling. We show that TGR5 activates a cAMP-PKA-STAT1 pathway to promote IFNλ3 and ISG15 expression, whereas FXR acts as a counter-regulatory brake that limits STAT1 activation and downstream IFN output.

## 2. Materials and Methods

### 2.1. Cell Culture and Treatments

IPEC-J2 porcine intestinal epithelial cells and MODE-K murine intestinal epithelial cells were maintained in DMEM (Gibco, C11995500BT, Grand Island, NY, USA) supplemented with 10% fetal bovine serum and 1% penicillin–streptomycin at 37 °C in a humidified 5% CO_2_ incubator. INT-777, fludarabine (FaraA), H89, SQ22536, and GW4064 were purchased from MedChemExpress (Monmouth Junction, NJ, USA) unless otherwise indicated. DMSO (Solarbio, ID9010, Beijing, China) was used as the vehicle control.

For TGR5 activation experiments, IPEC-J2 and MODE-K cells were treated with INT-777 at 50 or 100 μM for 24 h. To inhibit STAT1 phosphorylation, cells at approximately 60% confluence were pretreated with fludarabine (10 μM) for 24 h before exposure to INT-777 (100 μM, 24 h). To interrogate upstream signaling, cells were pretreated with the adenylate cyclase inhibitor SQ22536 (10 μM, 1 h) or the PKA inhibitor H89 (10 μM, 1 h) before INT-777 stimulation.

For FXR activation assays, cells were transfected with poly(I:C) (0.1 mg/mL) for 24 h and then treated with GW4064 (10 μM, unless otherwise indicated) for an additional 24 h. In receptor crosstalk experiments, cells were pretreated with GW4064 (10 μM, 24 h) followed by INT-777 (100 μM, 24 h). INT-777, GW4064, and pathway inhibitors were dissolved in DMSO before cell treatment, whereas poly(I:C) was dissolved in PBS. Cells treated with an equivalent concentration of DMSO or PBS were used as vehicle controls.

### 2.2. Cell Viability Assay

Cell viability following pharmacological treatment was evaluated using an MTT assay. IPEC-J2 and MODE-K cells were seeded in 96-well plates and allowed to adhere overnight. Cells were subsequently exposed to INT-777 (100 μM), GW4064 (10 and 20 μM), or FaraA (10 μM) for the indicated treatment durations. Combination treatments were performed using the same concentrations, treatment sequence, and exposure durations as those used in the corresponding signaling experiments. Vehicle-treated cells containing an equivalent final concentration of DMSO served as controls. Following treatment, MTT assays were performed according to the manufacturer’s instructions. Absorbance was measured using a microplate reader, and cell viability was calculated relative to the vehicle control. Three independent biological experiments were performed.

### 2.3. STAT1 Silencing

To examine the requirement for STAT1, IPEC-J2 cells were transfected with STAT1-targeting small interfering RNA using serum-free medium and Opti-MEM (Thermo Fisher Scientific, 31985062, Waltham, MA, USA) according to the manufacturer’s instructions. The STAT1 siRNA sequences were as follows: sense, 5′-GCGUAACCUUCAGGAUAAU-3′; antisense, 5′-AUUAUCCUGAAGGUUACGC-3′. After transfection, cells were stimulated with INT-777 (100 μM) for 24 h before downstream analyses. A non-targeting siRNA was used as the negative control.

### 2.4. RNA Extraction and RT-qPCR

Total RNA was isolated using RNA Isolater Total RNA Extraction Reagent (Vazyme, R401-01). cDNA was synthesized using the corresponding reverse-transcription kit according to the manufacturer’s instructions. Quantitative PCR was performed using CHANQ Blue Universal SYBR qPCR Master Mix (Vazyme, Q312-02) on a Bio-Rad real-time PCR system. Relative gene expression was calculated using the 2^−^^ΔΔCt^ method. GAPDH was used as the internal control for IPEC-J2 cells, and β-actin was used as the internal control for MODE-K cells. Primer sequences are listed in Table 1, and primer specificity was verified by melting curve analysis; all primer pairs showed a single specific amplification peak. β-actin was used as the internal reference gene for porcine IPEC-J2 cells; GAPDH was used as the internal reference gene for murine MODE-K cells.

### 2.5. Western Blot

Whole-cell lysates were prepared in RIPA buffer (Coolaber, SL1010, Beijing, China) containing 1 mM PMSF and clarified by centrifugation at 12,000× *g* for 10 min. Protein concentrations were determined using a BCA assay kit (Beyotime, P0010S, Shanghai, China). Equal amounts of protein (20 μg per sample) were denatured in loading buffer at 100 °C for 10 min, separated by 10% SDS-PAGE, and transferred onto PVDF membranes. Membranes were blocked in 5% nonfat milk or BSA for 2 h at room temperature and incubated overnight at 4 °C with primary antibodies against STAT1 (Proteintech, 66545), phospho-STAT1 (Tyr701, ABclonal, AP0135, Wuhan, China), GAPDH (ABclonal, AC027), IFNλ3 (Thermo Fisher Scientific, 103439), or ISG15 (Abcam, 308219, Cambridge, UK). After washing with TBST, membranes were incubated with HRP-conjugated secondary antibodies for 2 h at room temperature and visualized using ECL reagents (MeilunBio, MA0186, Dalian, China). The immunoblot signals were acquired using a ChemiDoc XRS+ System (Bio-Rad, 170-8265, Hercules, CA, USA) after ECL detection. Protein abundance was normalized to the corresponding loading control, and phosphorylated STAT1 levels were calculated relative to total STAT1 abundance.

### 2.6. Immunofluorescence Assay

To examine the subcellular localization of STAT1 and FXR, treated IPEC-J2 and MODE-K cells were fixed with immunostaining fixative (Beyotime, P0098) for 15 min at room temperature, washed three times with pre-chilled TBS, and permeabilized with Triton X-100 (Beyotime, P0096) for 10 min on ice. Cells were blocked with 5% BSA for 30 min at 37 °C and incubated overnight at 4 °C with anti-STAT1 antibody. After washing, cells were incubated with ABFLO 488-conjugated goat anti-mouse IgG (H + L) (ABclonal, AS076) to visualize STAT1. For FXR colocalization analyses, cells were subsequently incubated with anti-FXR antibody (Thermo Fisher Scientific, 16822) followed by Cy3-conjugated goat anti-rabbit IgG (H + L) (ABclonal, AS007). Nuclei were counterstained with DAPI (Thermo Fisher Scientific, D1306), and images were acquired by confocal microscopy.

### 2.7. Statistical Analysis

All experiments were independently repeated three times (n = 3 biological replicates). Data are presented as mean ± SD. Two-group comparisons were analyzed using an unpaired two-tailed Student’s *t*-test, whereas comparisons among three or more groups were analyzed by one-way ANOVA followed by an appropriate multiple-comparison test (GraphPad Prism 10.0). Differences were considered statistically significant when *p* < 0.05.

## 3. Results

### 3.1. TGR5 Activation Induces an Epithelial IFN/ISG Transcriptional Program

**Figure 1:**
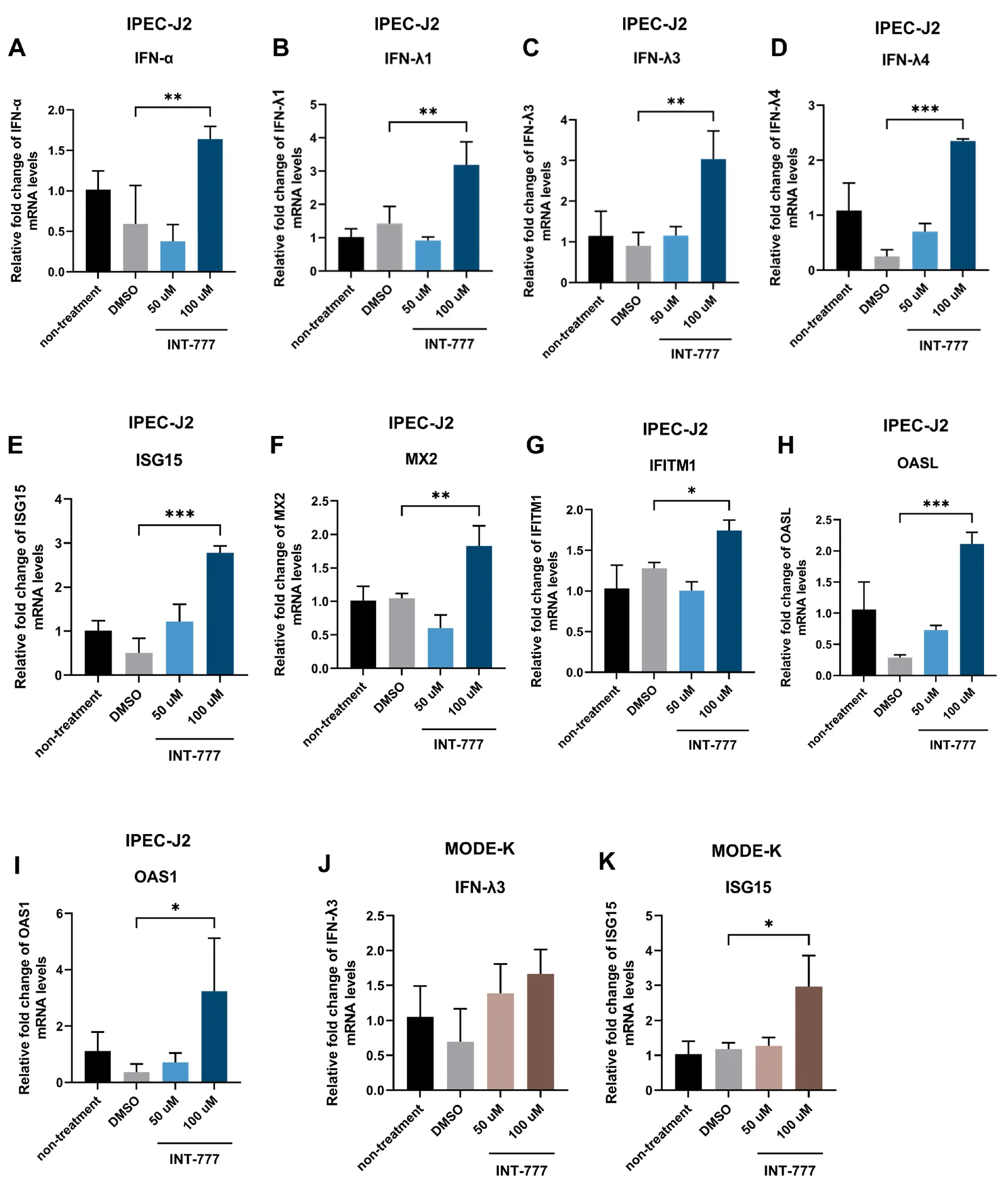
TGR5 activation induces IFN and ISG transcription in intestinal epithelial cells. IPEC-J2 and MODE-K cells were treated with INT-777 (50 or 100 μM) for 24 h. RT-qPCR analysis was performed to quantify IFN-α (**A**), IFN-λ1 (**B**), IFN-λ3 (**C**), IFN-λ4 (**D**), ISG15 (**E**), Mx2 (**F**), IFITM1 (**G**), OASL (**H**), and OAS1 (**I**) in IPEC-J2 cells, and IFN-λ3 (**J**) and ISG15 (**K**) in MODE-K cells. Gene expression was normalized to GAPDH or β-actin, as indicated. Data are presented as mean ± SD from three independent biological experiments (n = 3). * *p* < 0.05, ** *p* < 0.01, *** *p* < 0.001.

To determine whether TGR5 directly enhances epithelial interferon signaling, IPEC-J2 and MODE-K cells were stimulated with the selective TGR5 agonist INT-777, and the expression of representative IFNs and ISGs was examined by RT-qPCR. In IPEC-J2 cells, INT-777 increased the transcription of IFN-α and multiple type III IFNs, including IFNλ1, IFNλ3, and IFNλ4 (Figure 1A–D). INT-777 also elevated the expression of several ISGs, including ISG15, MX2, IFITM1, OASL, and OAS1 (Figure 1E–I). In MODE-K cells, where IFNλ3 is the principal epithelial type III IFN examined in this study, INT-777 similarly enhanced IFNλ3 and ISG15 transcription (Figure 1J,K). These data indicate that TGR5 activation is sufficient to trigger a broad epithelial IFN/ISG transcriptional program in both porcine and murine intestinal epithelial cells.

### 3.2. STAT1 Is Required for TGR5-Driven IFNλ3 and ISG15 Induction

**Figure 2:**
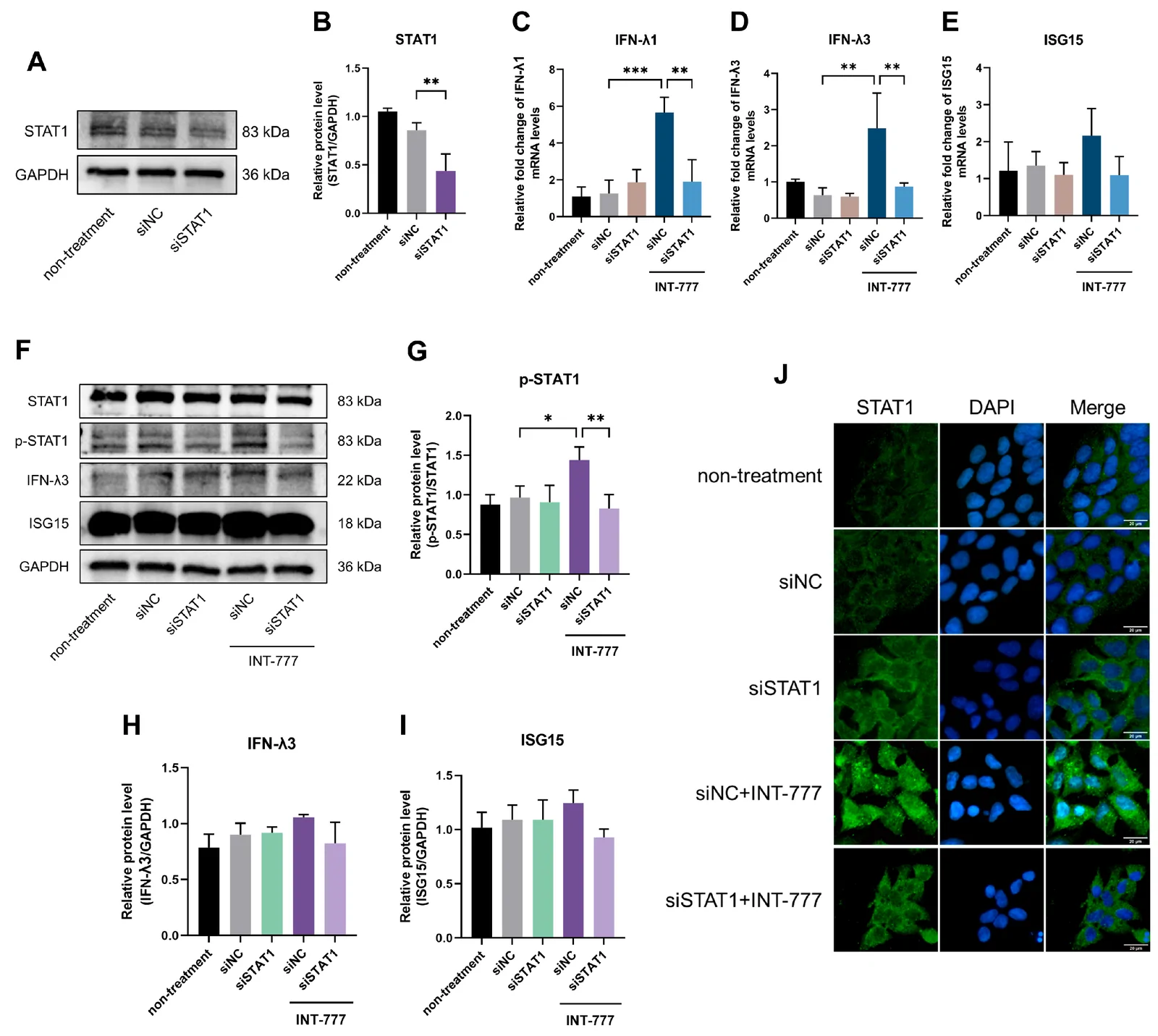
STAT1 is required for TGR5-induced IFN-λ3 and ISG15 expression in IPEC-J2 cells. STAT1 was silenced in IPEC-J2 cells by siRNA, and knockdown efficiency was confirmed by western blotting (**A**,**B**). Cells were then treated with INT-777 (100 μM) for 24 h, followed by RT-qPCR analysis of IFN-λ1 (**C**), IFN-λ3 (**D**), and ISG15 (**E**). Western blotting was used to detect p-STAT1, IFN-λ3, and ISG15 (**F**), with densitometric quantification shown in (**G**–**I**). (**J**) Representative confocal images showing STAT1 subcellular localization. Nuclei were stained with DAPI. Data are presented as mean ± SD from three independent biological experiments (n = 3). * *p* < 0.05, ** *p* < 0.01, *** *p* < 0.001.

Because STAT1 is a central node of IFN signaling, we next asked whether the TGR5 response depends on STAT1 activation. We focused on IFNλ3 and ISG15 because they showed robust induction and are highly relevant to epithelial antiviral immunity. In IPEC-J2 cells, small interfering RNA (siRNA)-mediated STAT1 knockdown markedly reduced INT-777-induced IFNλ1, IFNλ3, and ISG15 transcription (Figure 2A–E). Consistent with the transcriptional data, western blot analysis showed a reduction in IFNλ3 and ISG15 protein abundance following TGR5 activation in STAT1-depleted cells (Figure 2F–I). Although the differences did not reach statistical significance, both proteins exhibited a clear downward trend. Importantly, INT-777 increased STAT1 phosphorylation, whereas STAT1 knockdown attenuated this response (Figure 2F,G). Confocal imaging further showed that TGR5 activation promoted STAT1 accumulation in the nucleus, whereas STAT1 remained predominantly cytoplasmic in untreated and negative-control siRNA-transfected cells (Figure 2J).

**Figure 3:**
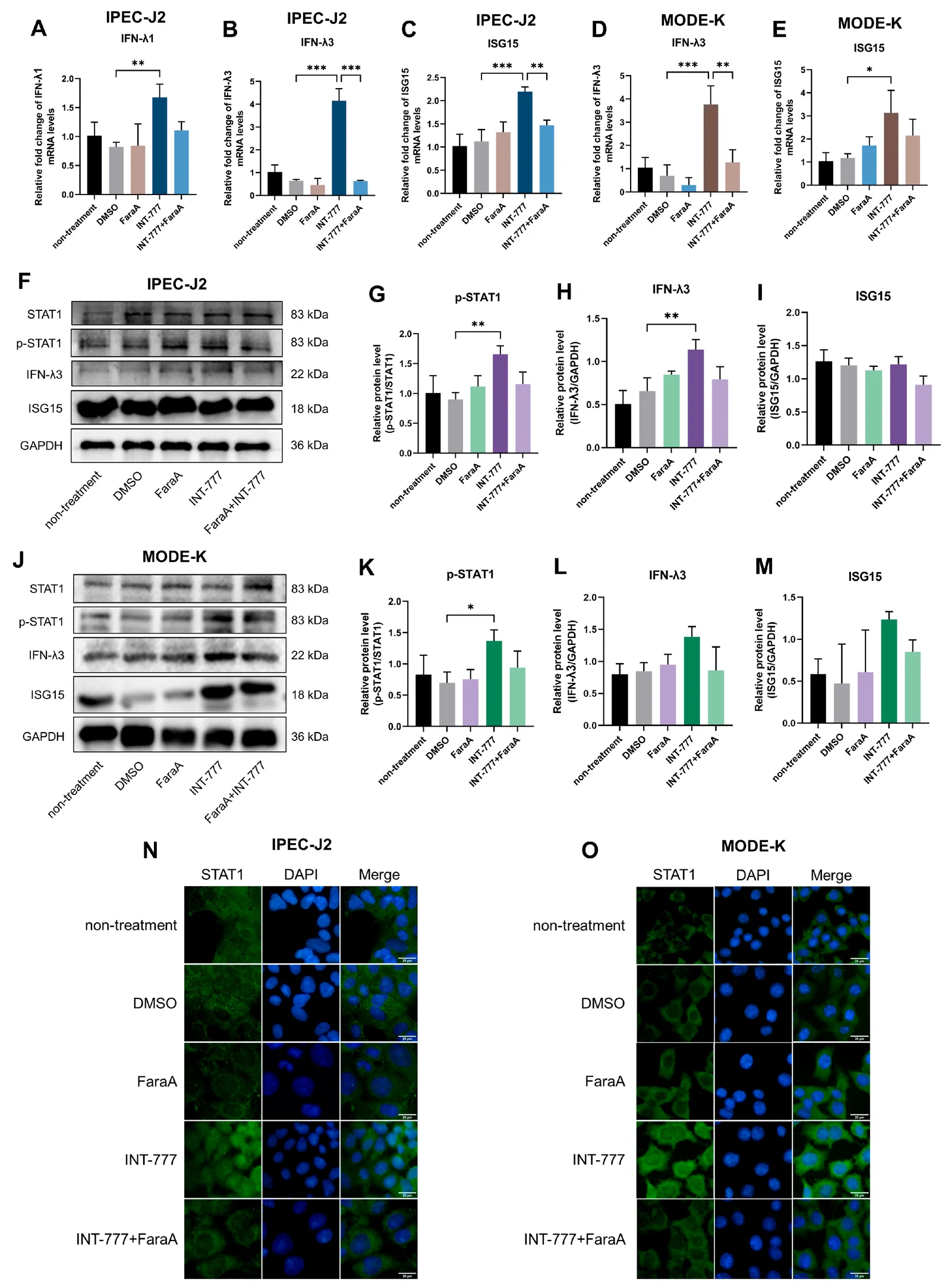
Inhibition of STAT1 phosphorylation suppresses TGR5-induced IFN-λ3 and ISG15 expression. IPEC-J2 and MODE-K cells were pretreated with fludarabine (FaraA, 10 μM) for 24 h and then stimulated with INT-777 (100 μM) for 24 h. RT-qPCR analysis was performed for IFN-λ1 (**A**), IFN-λ3 (**B**), and ISG15 (**C**) in IPEC-J2 cells, and IFN-λ3 (**D**) and ISG15 (**E**) in MODE-K cells. Western blotting and densitometric analyses of p-STAT1, IFN-λ3, and ISG15 are shown for IPEC-J2 cells (**F**–**I**) and MODE-K cells (**J**–**M**). (**N**,**O**) Representative confocal images of STAT1 localization in IPEC-J2 (**N**) and MODE-K (**O**) cells. Nuclei were stained with DAPI. The relative phosphorylation level of STAT1 was calculated as (p-STAT1/loading control)/(total STAT1/loading control). Data are presented as mean ± SD from three independent biological experiments (n = 3). * *p* < 0.05, ** *p* < 0.01, *** *p* < 0.001.

To further test whether STAT1 phosphorylation is functionally required, IPEC-J2 and MODE-K cells were treated with the STAT1 phosphorylation inhibitor fludarabine (FaraA) before INT-777 stimulation. FaraA significantly suppressed TGR5-induced IFNλ1, IFNλ3, and ISG15 transcription, as well as IFNλ3 and ISG15 protein expression, in both cell types (Figure 3A–M). In parallel, FaraA blocked TGR5-driven nuclear accumulation of STAT1 (Figure 3N,O). Together, these findings place STAT1 phosphorylation and nuclear translocation upstream of the TGR5-mediated induction of IFNλ3 and ISG15.

### 3.3. TGR5 Engages a cAMP-PKA-STAT1 Pathway in Intestinal Epithelial Cells

**Figure 4:**
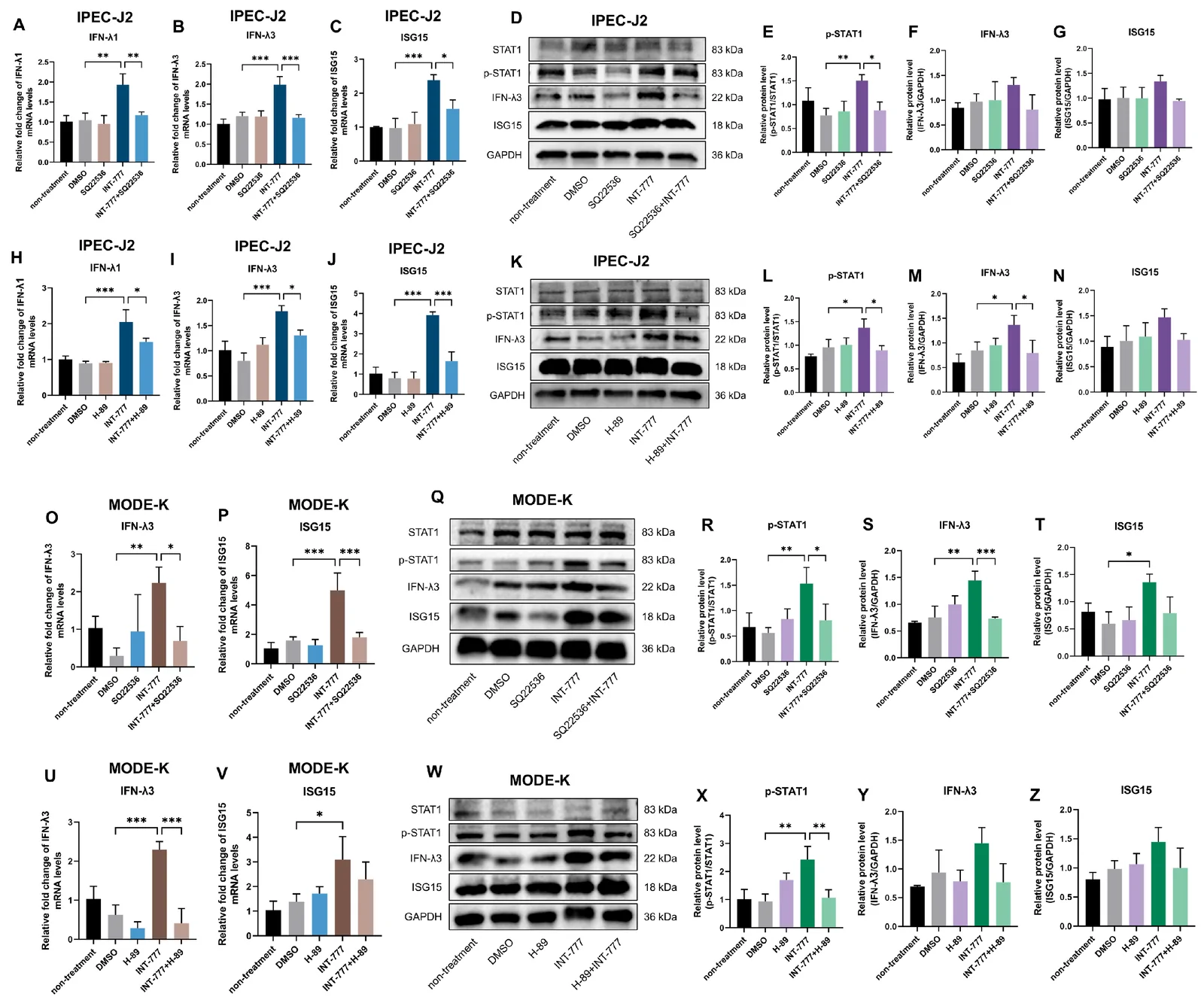
TGR5 promotes IFN-λ3 and ISG15 expression through cAMP-PKA-dependent STAT1 activation. IPEC-J2 and MODE-K cells were pretreated with the adenylate cyclase inhibitor SQ22536 (10 μM, 1 h) or the PKA inhibitor H89 (10 μM, 1 h), followed by stimulation with INT-777 (100 μM, 24 h). After SQ22536 treatment, RT-qPCR analysis was performed for IFN-λ1 (**A**), IFN-λ3 (**B**), and ISG15 (**C**) in IPEC-J2 cells, and IFN-λ3 (**O**) and ISG15 (**P**) in MODE-K cells. Western blotting and densitometric analyses of p-STAT1, IFN-λ3, and ISG15 are shown in IPEC-J2 cells (**D**–**G**) and MODE-K cells (**Q**–**T**). After H89 treatment, RT-qPCR analysis was performed for IFN-λ1 (**H**), IFN-λ3 (**I**), and ISG15 (**J**) in IPEC-J2 cells, and IFN-λ3 (**U**) and ISG15 (**V**) in MODE-K cells. Western blotting and densitometric analyses are shown in IPEC-J2 cells (**K**–**N**) and MODE-K cells (**W**–**Z**). Data are presented as mean ± SD from three independent biological experiments (n = 3). * *p* < 0.05, ** *p* < 0.01, *** *p* < 0.001.

TGR5 is canonically coupled to adenylate cyclase activation and downstream cAMP-PKA signaling [23]. We therefore examined whether this pathway mediates STAT1 activation downstream of TGR5. In both IPEC-J2 (Figure 4A–G) and MODE-K (Figure 4O–T) cells, pharmacological inhibition of adenylate cyclase with SQ22536 reduced INT-777-induced IFNλ3 and ISG15 transcription and diminished the expression of the corresponding proteins. Similar effects were observed after inhibition of PKA with H89 in IPEC-J2 (Figure 4H–N) and MODE-K (Figure 4U–Z) cells. In both cell types, blockade of adenylate cyclase or PKA also reduced TGR5-driven STAT1 phosphorylation. These data support a model in which TGR5 activation was associated with increased STAT1 phosphorylation and enhanced IFNλ3/ISG15 expression. Pharmacological inhibition of cAMP–PKA signaling reduced these responses, suggesting that cAMP–PKA signaling contributes to STAT1 activation downstream of TGR5 stimulation.

### 3.4. FXR Activation Suppresses Poly(I:C)-Induced IFNλ3 and ISG15 Transcription

**Figure 5:**
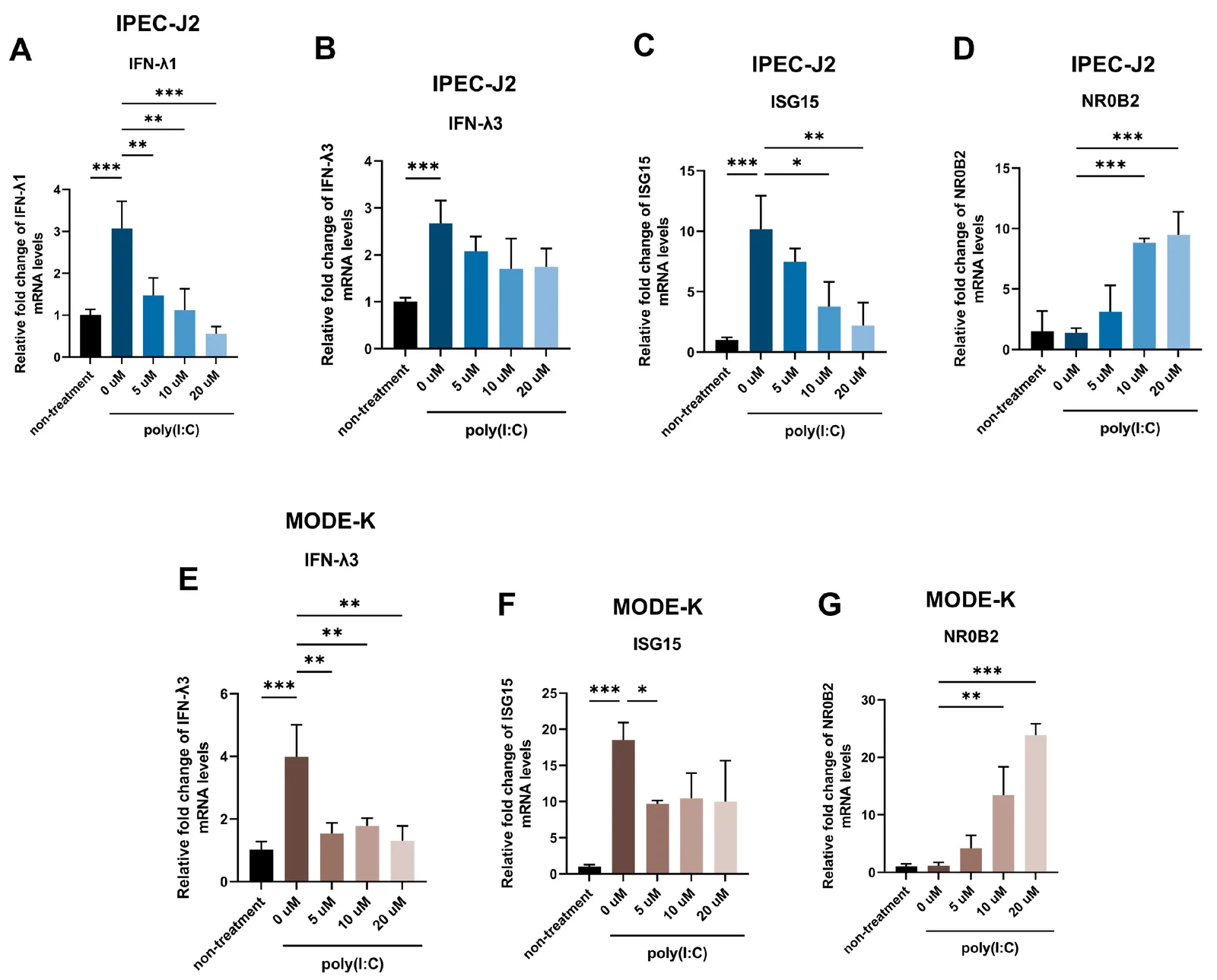
FXR activation inhibits IFN-λ3 and ISG15 transcription in intestinal epithelial cells. IPEC-J2 and MODE-K cells were transfected with poly(I:C) (0.1 mg/mL) for 24 h and then treated with the FXR agonist GW4064 (10 μM) for 24 h. RT-qPCR analysis was performed for IFN-λ1 (**A**), IFN-λ3 (**B**), ISG15 (**C**), and NR0B2 (**D**) in IPEC-J2 cells, and IFN-λ3 (**E**), ISG15 (**F**), and NR0B2 (**G**) in MODE-K cells. Data are presented as mean ± SD from three independent biological experiments (n = 3). * *p* < 0.05, ** *p* < 0.01, *** *p* < 0.001.

Because bile acids can activate multiple receptors with potentially divergent immune consequences, we next evaluated the effect of FXR on epithelial IFN signaling. Poly(I:C), a synthetic double-stranded RNA mimic, robustly induced IFNλ1, IFNλ3, and ISG15 transcription in IPEC-J2 cells and induced IFNλ3 and ISG15 transcription in MODE-K cells. Subsequent treatment with the FXR agonist GW4064 reduced poly(I:C)-induced IFNλ1, IFNλ3, and ISG15 expression in a dose-dependent manner in IPEC-J2 (Figure 5A–C) and MODE-K (Figure 5E,F) cells. The FXR target gene NR0B2 was induced in parallel (Figure 5D,G), confirming effective FXR activation. These results indicate that FXR imposes a suppressive effect on epithelial IFN transcriptional responses.

### 3.5. FXR Counteracts TGR5-Driven STAT1 Activation and Downstream Gene Expression

**Figure 6:**
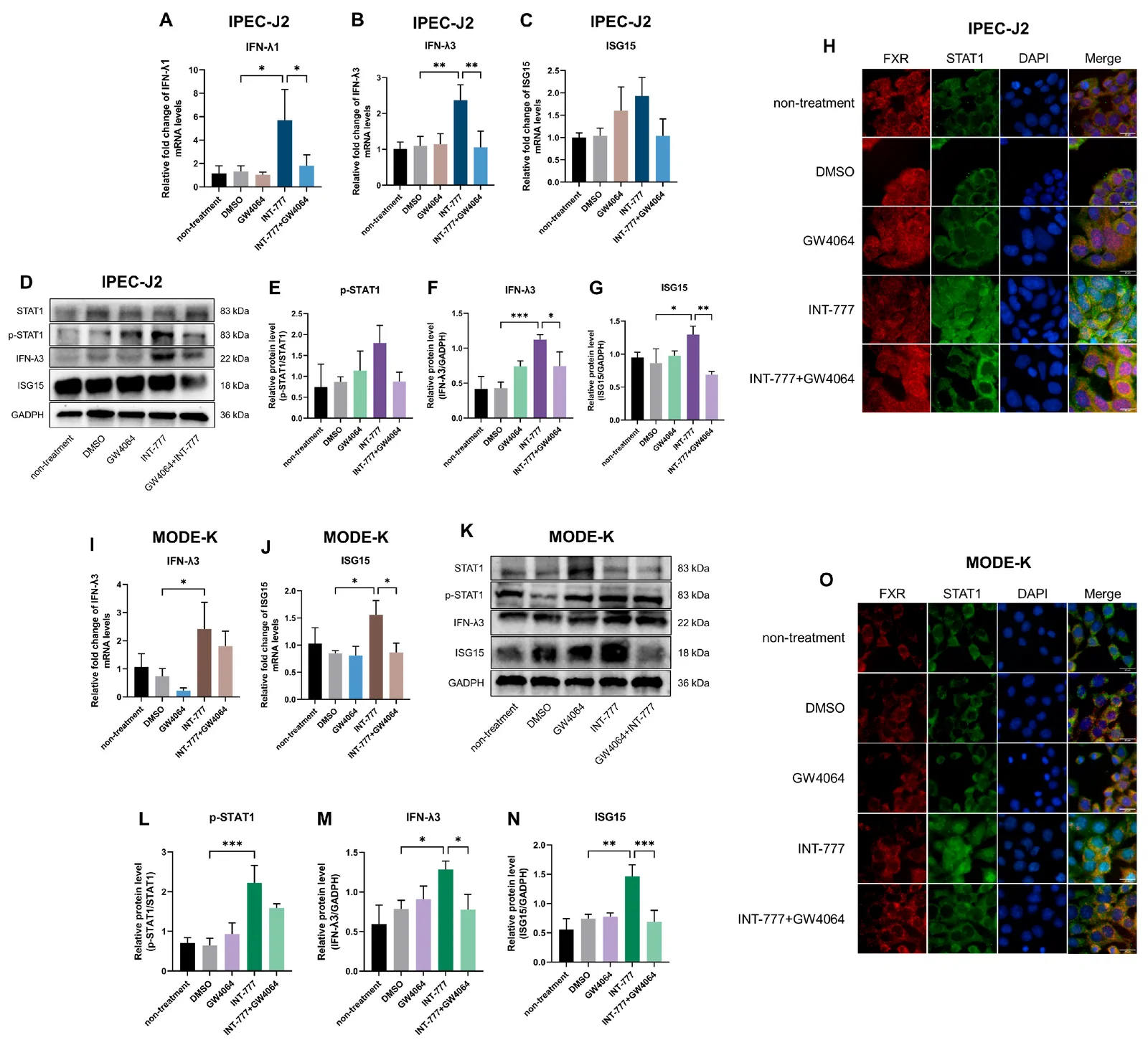
FXR activation antagonizes TGR5-driven IFN-λ3 and ISG15 expression. IPEC-J2 and MODE-K cells were pretreated with GW4064 (10 μM, 24 h) and then stimulated with INT-777 (100 μM, 24 h). RT-qPCR analysis was performed for IFN-λ1 (**A**), IFN-λ3 (**B**), and ISG15 (**C**) in IPEC-J2 cells, and IFN-λ3 (**I**) and ISG15 (**J**) in MODE-K cells. Western blotting and densitometric analyses of p-STAT1, IFN-λ3, and ISG15 are shown for IPEC-J2 cells (**D**–**G**) and MODE-K cells (**K**–**N**). (**H**,**O**) Representative confocal images showing the subcellular localization of STAT1 and FXR in IPEC-J2 (**H**) and MODE-K (**O**) cells. Nuclei were stained with DAPI. Data are presented as mean ± SD from three independent biological experiments (n = 3). The scale bars in panels H and O represent 20 μm.* *p* < 0.05, ** *p* < 0.01, *** *p* < 0.001.

Having established opposite effects of TGR5 and FXR on epithelial IFN output, we next asked whether FXR antagonizes the TGR5 response through STAT1. In both IPEC-J2 (Figure 6A–G) and MODE-K (Figure 6I–N) cells, activation of FXR significantly reduced INT-777-induced IFNλ3 and ISG15 transcription and decreased the corresponding protein levels. Notably, FXR activation also attenuated TGR5-driven STAT1 phosphorylation. Confocal microscopy further showed differences in the subcellular distribution of STAT1 among different treatments. Compared with TGR5 activation, FXR activation was associated with reduced apparent nuclear accumulation of STAT1 and increased cytoplasmic localization (Figure 6H,O). These observations provide supportive evidence that FXR activation may influence STAT1 subcellular distribution; however, the immunofluorescence images were interpreted qualitatively and were not used as a quantitative measurement of nuclear/cytoplasmic fluorescence intensity. These findings suggest functional cross-talk between FXR and STAT1 signaling.

### 3.6. Pharmacological Treatments Do Not Significantly Reduce Intestinal Epithelial Cell Viability

**Figure 7:**
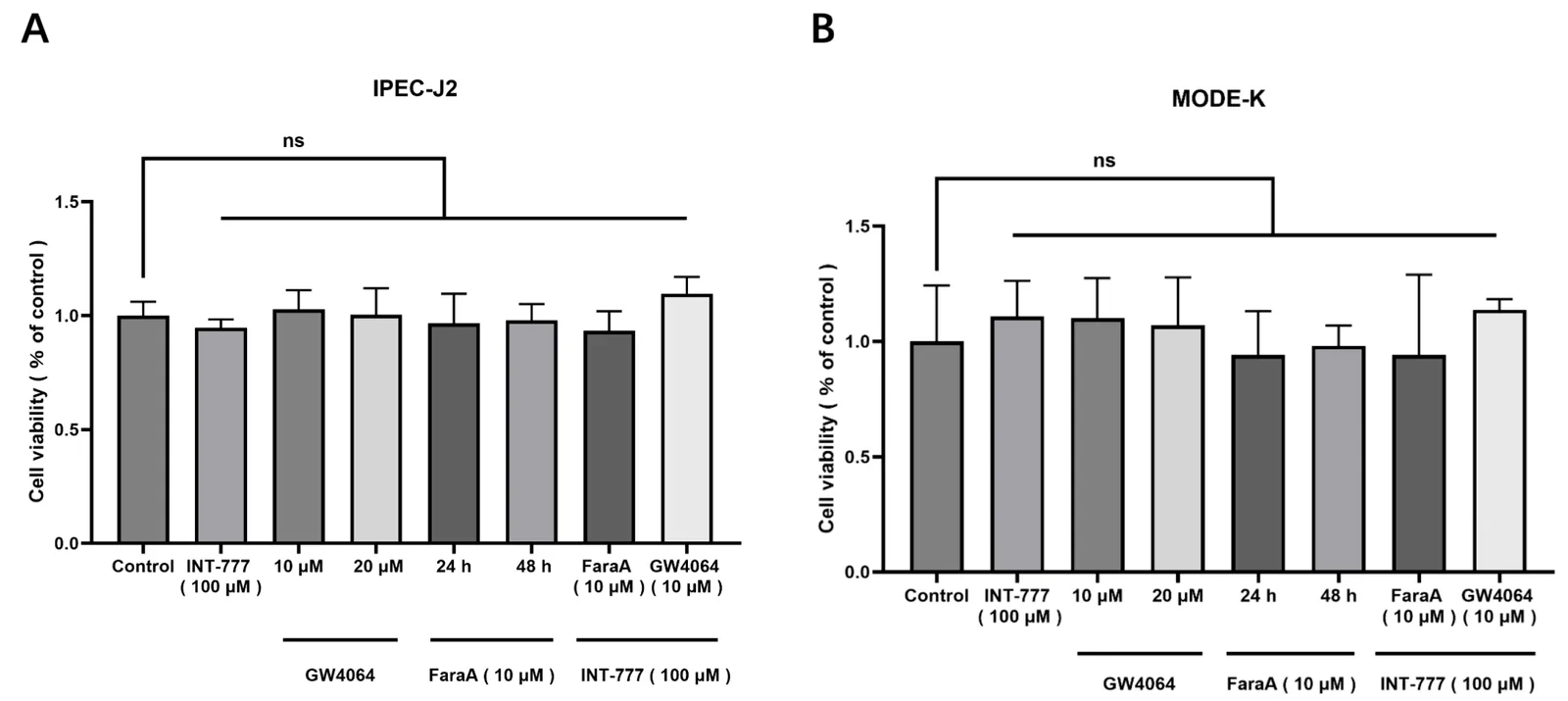
Effects of pharmacological treatments on the viability of intestinal epithelial cells. IPEC-J2 (**A**) and MODE-K (**B**) cells were treated with INT-777 (100 μM), GW4064 (10 or 20 μM), or fludarabine (FaraA, 10 μM) for the indicated exposure durations. Combination treatments with FaraA (10 μM) plus INT-777 (100 μM) or GW4064 (10 μM) plus INT-777 (100 μM) were performed using the same treatment conditions as those used in the corresponding signaling experiments. Cell viability was evaluated using an MTT assay and expressed relative to the vehicle control. Data are presented as mean ± SD from three independent biological experiments (n = 3). Statistical comparisons were performed using one-way ANOVA followed by Dunnett’s multiple-comparisons test versus the vehicle control. ns, not significant.

To determine whether the pharmacological treatment conditions used in this study affected cell viability, MTT assays were performed in IPEC-J2 and MODE-K cells using the same drug concentrations and exposure durations employed in the signaling experiments. Treatment with INT-777 (100 μM), GW4064 (10 or 20 μM), or fludarabine (FaraA, 10 μM) for 24 or 48 h did not significantly reduce cell viability compared with the corresponding vehicle control in either cell line (Figure 7A,B). Likewise, the combined treatment conditions, including FaraA (10 μM) plus INT-777 (100 μM) and GW4064 (10 μM) plus INT-777 (100 μM), did not result in a significant reduction in cell viability. These results indicate that the pharmacological treatment conditions used in this study did not significantly impair MTT-based metabolic cell viability in either IPEC-J2 or MODE-K cells.

## 4. Discussion

This study identifies a bile acid receptor signaling rheostat that tunes epithelial interferon output through opposite control of STAT1. Across porcine IPEC-J2 and murine MODE-K cells, TGR5 activation enhanced IFNλ3 and ISG15 expression, whereas FXR activation exerted the opposite effect. Mechanistically, TGR5 promoted STAT1 phosphorylation and nuclear accumulation through a cAMP-PKA-dependent pathway, while FXR restrained this response and limited downstream gene induction. Thus, the key implication of this work is not simply that bile acid receptors modulate IFN signaling, but that distinct bile acid-sensing pathways can bias intestinal epithelial cells toward either an IFN-amplified or an IFN-restrained state. Our findings extend growing evidence that bile acids participate in antiviral defense beyond their metabolic functions. Previous studies have shown that several bile acid species can restrict the replication of enteric and non-enteric viruses while enhancing interferon responses [21,24]. In porcine epithelial systems, lithocholic acid and hyodeoxycholic acid have been linked to TGR5-dependent induction of interferon-related genes and suppression of coronavirus replication [19,22,25]. The present work refines this concept by positioning TGR5 as a direct upstream regulator of an epithelial STAT1-centered transcriptional program. Notably, the strongest responses in our system involved IFNλ3 and ISG15, which is consistent with the dominant role of type III IFNs in mucosal antiviral defense and with the importance of ISG15 as an epithelial antiviral effector [14,15].

This preference for IFNλ3 is biologically plausible in the context of epithelial immunity. Type I and type III IFNs are central antiviral cytokines that enhance host recognition of, responsiveness to, and control of invading pathogens [26,27]. Following the sensing of viral pathogen-associated molecular patterns by pattern-recognition receptors, signaling pathways involving cGAS-STING, Toll-like receptors, and RIG-I-like receptors activate IRF3 and drive the production of type I and type III IFNs [11,28]. Among these IFN families, type III IFNs are particularly important at epithelial barriers, where they reinforce barrier integrity and strengthen host defense against parasites, bacteria, and viruses [11,29,30]. Intestinal epithelial cells can also produce basal type III IFNs in the absence of overt infection, thereby maintaining low-level ISG expression and constitutive antiviral readiness [31]. Importantly, not all type III IFNs appear to contribute equally to this homeostatic state. Compared with IFNλ1, IFNλ2/3 make the dominant contribution to basal antiviral protection in the intestinal epithelium [11]. In human intestinal epithelial systems, IFNλ2/3 are particularly important for restricting the spread of rotavirus, mammalian reovirus, vesicular stomatitis virus, and vaccinia virus [11,32,33]. Against this background, the preferential induction of IFNλ3 observed here is consistent with a physiologically relevant epithelial antiviral program.

Within the JAK-STAT pathway, STAT1 represents a critical signaling checkpoint for downstream IFN signaling and ISG induction [34]. Several viruses have evolved mechanisms to disrupt this step. For example, the thymidine kinase protein of pseudorabies virus interferes with JAK1-STAT1 complex formation through interaction with JAK and STAT1, thereby inhibiting STAT1 phosphorylation [35]. Orf virus also directly targets STAT1 to suppress both its phosphorylation and nuclear translocation [36]. In addition, the PEDV nucleocapsid protein promotes STAT1 acetylation, which prevents phosphorylation and impairs its nuclear trafficking [37]. These observations collectively underscore the importance of STAT1 phosphorylation in antiviral IFN signaling. Consistent with this concept, our data show that TGR5 activation promotes STAT1 phosphorylation and nuclear translocation in both IPEC-J2 and MODE-K cells, thereby enhancing IFNλ3 and ISG15 expression. These findings place STAT1 at the center of the epithelial TGR5 response and identify it as a key signaling node linking bile acid receptor activation to epithelial interferon output.

Interestingly, the relationship between TGR5 and IFN signaling may be bidirectional. Xiong and colleagues reported that IFN signaling itself can upregulate TGR5 expression [38]. In that study, viral infection induced TGR5 expression in an IFN/STAT1-dependent manner, whereas TGR5 in turn amplified type I IFN signaling through AKT-mediated IRF3 activation [38]. TGR5 overexpression also suppressed the replication of VSV, NDV, and HSV-1 [38]. Together with our findings, these observations support the concept that TGR5 is not merely a bile acid sensor, but an active amplifier of epithelial antiviral interferon circuitry.

A mechanistic advance of the present study is the identification of a cAMP-PKA-STAT1 axis downstream of TGR5 in intestinal epithelial cells. TGR5 is known to couple to adenylate cyclase, elevate intracellular cAMP, and activate PKA [39,40]. This pathway has been implicated in diverse biological settings, including insulin secretion and anti-inflammatory regulation. In pancreatic β-cells, TGR5 activation stimulates insulin secretion through cAMP-PKA signaling [41,42]. In immune cells, TGR5-cAMP-PKA signaling suppresses NF-κB activation and dampens inflammatory cytokine production [43,44]. In dendritic cells, for example, TGR5 signaling reduces phosphorylation of p65 and IκBα and lowers the secretion of IL-1β, IL-6, TNF-α, and IL-12p70 [44]. Beyond NF-κB, TGR5-cAMP-PKA signaling has also been shown to inhibit NLRP3 inflammasome activation and alleviate influenza virus-induced lung inflammation [45]. In microglia, the bile acid TUDCA acts through TGR5 to suppress pro-inflammatory markers while promoting anti-inflammatory programs [46]. In antiviral contexts, TGR5-cAMP signaling has likewise been linked to induction of IFNβ and ISG15 and to suppression of PEDV replication [25]. Our results extend these observations by showing that, in intestinal epithelial cells, TGR5 promotes IFNλ3 and ISG15 expression while enhancing STAT1 phosphorylation through cAMP-PKA signaling. Supporting this interpretation, cAMP has been reported to promote STAT1 phosphorylation in murine splenocytes, whereas the PKA inhibitor H89 suppresses Ca^2+^-PKC-induced STAT1 phosphorylation [47]. Taken together, these findings support cAMP-PKA signaling as an important upstream regulator of STAT1 activation in the epithelial TGR5 response.

Although our findings support the involvement of cAMP–PKA-dependent STAT1 activation in TGR5-mediated interferon responses, the precise hierarchy of this signaling network remains to be fully established. Previous studies have demonstrated that TGR5 can regulate antiviral responses through AKT–IRF3 signaling, whereas STAT1 may also regulate TGR5 expression during infection-induced interferon responses [39]. Therefore, the relationship between TGR5 and STAT1 may involve reciprocal regulation rather than a simple linear pathway. In addition, because the present study used INT-777 to pharmacologically activate endogenous TGR5 under non-infectious conditions, the proposed cAMP–PKA–STAT1 signaling axis should be interpreted as a functional model that requires further validation using genetic approaches and viral infection models.

Equally important, our data identify FXR as a counter-regulatory brake on epithelial IFN signaling. TGR5 activation has been reported to promote virus-induced IFNB1 transcription, whereas FXR activation does not exert the same effect [21]. More recently, FXR was shown to weaken the antiviral efficacy of bile acids by suppressing IFN pathway activation [20]. In that study, FXR activation inhibited poly(I:C)-induced Ifnb1 transcription and reduced the antiviral activity of CDCA through negative regulation of interferon signaling [20]. Our results extend this concept beyond a type I IFN-centered context by showing that, in IPEC-J2 and MODE-K cells, FXR activation suppresses IFNλ1, IFNλ3, and the downstream effector ISG15. These findings suggest that STAT1 represents a convergent point of antagonism between TGR5 and FXR.

The mechanism by which FXR restrains epithelial IFN signaling remains to be fully defined, but the available evidence supports cross-talk with canonical IFN regulators. Liang and colleagues showed that FXR interacts strongly with IRF3 and that viral infection further enhances this interaction, thereby suppressing IRF3 transcriptional activity and downstream IFN signaling [20]. Their interaction screen further identified STAT1, in addition to IRF3, as a potential FXR-associated signaling component [20]. In our study, FXR activation inhibited STAT1 phosphorylation and reduced TGR5-induced IFNλ3 and ISG15 expression in both IPEC-J2 and MODE-K cells. Confocal imaging further showed altered cytoplasmic distribution and reduced apparent nuclear accumulation of STAT1 after FXR activation. These observations support a potential regulatory relationship between FXR signaling and STAT1 localization; however, they should be interpreted as supportive evidence because quantitative analysis of nuclear-to-cytoplasmic fluorescence intensity was not performed. Accordingly, the most defensible interpretation of our data is that FXR limits STAT1 activation and localization in a manner consistent with, although not yet definitively proving, a direct FXR-STAT1 interaction.

Several points should be considered when interpreting these findings. First, this study was performed in epithelial cell lines, and in vivo validation in the porcine intestine will be important to establish physiological relevance under the complex bile acid conditions present in animals. Second, most pathway inference in this work relies on pharmacological perturbation, so complementary receptor loss-of-function or rescue experiments would further strengthen causality. Third, although our data support STAT1 as the key signaling node downstream of TGR5 and under FXR antagonism, they do not exclude additional contributions from IRF3, STAT2, IRF9, or other transcriptional regulators. Finally, the study focuses on interferon pathway readouts rather than direct viral challenge in the same experimental series; therefore, the antiviral implications should be interpreted as mechanistically informed rather than directly demonstrated here.

Species-specific bile acid composition may also influence the biological relevance of this pathway. Hyocholic acid and hyodeoxycholic acid species and their derivatives account for nearly 80% of total plasma bile acids in pigs, but only approximately 1% in humans and rats [48]. This marked difference may have important consequences for receptor engagement in the porcine intestine. Notably, hyocholic acid can function both as a TGR5 agonist and an FXR antagonist, and has been shown to promote glucagon-like peptide-1 secretion through simultaneous activation of TGR5 and inhibition of FXR [49,50]. These observations further support the physiological plausibility of receptor opposition between TGR5 and FXR and suggest that the porcine bile acid milieu may favor signaling states that are not fully captured in standard non-porcine models.

A limitation of the present study is that TGR5 activation was achieved using the pharmacological agonist INT-777 at a relatively high concentration. Although INT-777 is widely used as a TGR5 agonist, potential off-target effects cannot be completely excluded. Therefore, our findings should be interpreted as pharmacological evidence supporting the involvement of TGR5 signaling in STAT1-dependent interferon regulation. Future studies using genetic approaches, including TGR5 knockdown or knockout models, will be necessary to further validate the receptor-specific contribution of TGR5. Furthermore, integrating transcriptomic profiling, computational network analysis, and receptor-interaction prediction approaches may further identify additional regulatory nodes involved in TGR5–FXR-mediated modulation of epithelial interferon responses.

## 5. Conclusions

Our study identifies TGR5 and FXR as opposing regulators of STAT1-dependent interferon signaling in intestinal epithelial cells. Mechanistically, TGR5 enhances STAT1 phosphorylation and nuclear translocation through a cAMP-PKA-dependent pathway, thereby promoting IFNλ3 and ISG15 expression, whereas FXR restrains STAT1 activation and dampens downstream interferon output. These findings define a bile acid receptor-regulated pathway that calibrates epithelial innate antiviral responses and expand current understanding of how metabolic sensing interfaces with mucosal immunity. Given the central role of epithelial barriers in host defense, the TGR5-FXR-STAT1 axis identified here may provide a useful framework for future antiviral and immunomodulatory intervention strategies.

## Figures and Tables

**Table 1:** Primers used in this study.

Primer Name	Forward Primer Sequence (5′-3′)	Reverse Primer Sequence (5′-3′)
Pig-IFN-α	GCCTCCTGCACCAGTTCTACA	TGCATGACAGGCTTCCA
Pig-IFNλ1	GGTGCTGGCGACTGTGATG	GATTGGAACTGGCCCATGTG
Pig-IFNλ3	CCTTCAAGAGGGCCAAGGATGCC	GTGAAGGGGCTGGTCCAGGC
Pig-IFNλ4	GCTATGGGACTGTGGGTCTT	AGGGAGCGGTAGTGAGAGAG
Pig-OAS1	GCCTGTGATTCTGGACCCGGCTGA	CGACACCTTCCAGGATCCCACCG
Pig-OASL	TCCCTGGGAAGAATGTGCAG	CCCTGGCAATGGTGATGAC
Pig-IFITM1	TGCCTCCACCGCCAAGT	GTGGCTCCGATGCTCAGAAT
Pig-ISG15	AGCATGGTCCTGTTGATGGTG	CAGAAATGGTCAGCTTGCACG
Pig-MX2	TTCACTCGCATCCGCACTTCAG	AGCTCCTCTGTCGCACTATGG
Pig-NR0B2	AGTGCTGCCTGGAGTCCTTA	GATGTGGGAGGAGGCATAGA
Pig-β-actin	AATCCTGCGGCATCCACGAAAC	GTGTTGGCGTAGAGGTCCTTGC
Mouse-GAPDH	TGTGTCCGTCGTGGATCTGA	GCTTCACCACCTTCTTGAT
Mouse-IFNλ3	CCAGTGGAAGCAAAGGATTGCC	GCACCTCATGTCCTTCTCAAGC
Mouse-ISG15	CATCCTGGTGAGGAACGAAAGG	CTCAGCCAGAACTGGTCTTCGT
Mouse-NR0B2	TGGGTCCCAAGGAGTATGC	GCTCCAAGACTTCACACAGTG

## Data Availability

The original contributions presented in this study are included in the article. Further inquiries can be directed to the corresponding author.

## Abbreviations

TGR5	Takeda G-protein-coupled receptor 5
FXR	Farnesoid X receptor
IFN	Interferon
ISGs	Interferon-stimulated genes
STAT	Signal transducer and activator of transcription
cAMP	Cyclic adenosine monophosphate
PKA	Protein kinase A
JAK	Janus kinase
OAS	Oligoadenylate synthase
IFITM	Interferon-induced transmembrane
MX	Myxovirus-resistant
IPEC-J2	Intestinal porcine epithelial cells
MODE-K	Murine enterocyte cells
cGAS	Cyclic GMP-AMP synthase
STING	Stimulator of interferon genes
AKT	Protein kinase B
IRF	Interferon regulatory factor
VSV	Vesicular stomatitis virus
NDV	Newcastle disease virus
HSV	Herpes simplex virus
NF-κB	Nuclear factor-kappa B
IL	Interleukin
TNF-α	Tumor necrosis factor-α
NLRP3	NOD-like receptor protein 3
TUDCA	Tauro-ursodeoxycholic acid
PEDV	Porcine epidemic diarrhea virus
CDCA	Chenodeoxycholic acid
